# Identification of antigenic proteins from the venom of Malaysian snakes using immunoprecipitation assay and tandem mass spectrometry (LC-MS/MS)

**DOI:** 10.1016/j.heliyon.2024.e37243

**Published:** 2024-09-01

**Authors:** Preetha Rajendiran, Rakesh Naidu, Iekhsan Othman, Syafiq Asnawi Zainal Abidin

**Affiliations:** a Jeffrey Cheah School of Medicine of Health Sciences, Jalan Lagoon Selatan, Monash University Malaysia, 47500, Bandar Sunway, Selangor Darul Ehsan, Malaysia; b Proteomics and Metabolomics Platform, Jeffrey Cheah School of Medicine and Health Sciences, Jalan Lagoon Selatan, Monash University Malaysia, 47500, Bandar Sunway, Selangor Darul Ehsan, Malaysia

**Keywords:** Antivenom, Proteomics, Malaysian venomous snake, Elapidae, Crotalinae

## Abstract

Snake envenomation poses a significant risk to Malaysians and country visitors. Malaysia witnesses an estimated 650 snake bites per 100,000 population annually. The primary treatment for snake envenomation involves administering antivenom derived from horses, despite its drawbacks, such as anaphylactic reactions and serum sickness. Identifying the venom proteome is crucial for understanding and predicting the clinical implications of envenomation and developing effective treatments targeting specific venom proteins. In this study, we employ an immunoprecipitation assay followed by LC-MS/MS to identify antigenic proteins in five common venomous snakes in Malaysia compassing of two families which are pit vipers, (*Calloselasma rhodostoma* and *Cryptelytrops purpureomaculatus*) and cobras (*Ophiophagus hannah, Naja kaouthia,* and *Naja sumatrana*). The immunoprecipitation assay utilises a 2 % agarose gel, allowing antigenic proteins to diffuse and bind with antibodies in the antivenom. The antivenom utilised in this research was procured from the Queen Saovabha Memorial Institute (QSMI), Thailand, including king cobra antivenom (KCAV), cobra antivenom (CAV), Malayan pit viper antivenom (MPAV), Russell's viper antivenom (RPAV), hematopolyvalent antivenom (HPAV), neuropolyvalent antivenom (NPAV), banded krait antivenom (BKAV), and Malayan krait antivenom (MKAV). The protein identified through these interactions which are exclusive to the cobras are three-finger toxins (3FTXs) while snake C-type lectins (Snaclecs) are unique to the pit vipers. Common protein that are present in both families are L-amino acid oxidase (LAAO), Phospholipase A_2_ (PLA_2_), and snake venom metalloproteinase (SVMP). Identifying these proteins is vital for formulating a broad-spectrum antivenom applicable across multiple species.

## Highlights

• Unique cobra proteins include 3FTXs, while pit vipers have Snaclecs.
• Common venom proteins detected across both snake families are LAAO, PLA_2_, and SVMP.
• The study reveals key proteins that could enable broader antivenom development.
• Novel findings offer potential improvements in treating diverse snakebites.
• These insights pave the way for more effective universal antivenom.

## Graphical abstract

**Figure:**
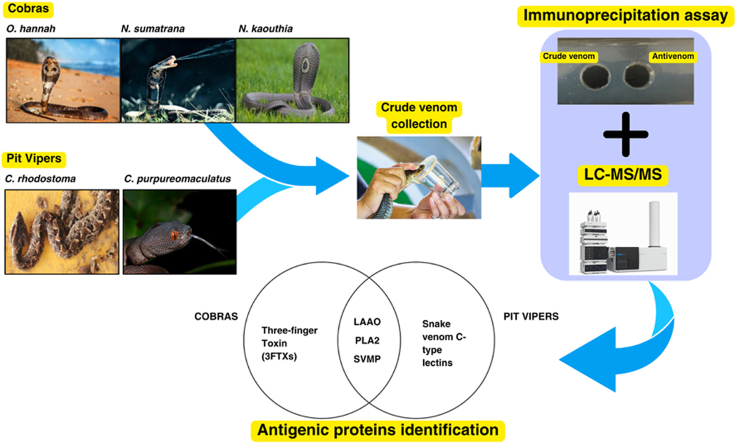

## 1 Introduction

Venomous snakes pose a significant threat to human health worldwide, with the World Health Organization (WHO) reclassifying them as one of the world's neglected tropical diseases in 2017 [1]. WHO reports approximately 81,000 to 138,000 deaths worldwide annually due to snakebite envenomation, with a high incidence of snakebites recorded in the Southeast Asian region [1]. Envenomation can happen when venomous snake bites or when venom is sprayed into the eyes of the victim by snakes that possess the capability to spray their venom as an act of defence [2]. In Malaysia, cobras and pit vipers are recognized as medically significant snakes [2], and these species are commonly found throughout the land region of peninsula Malaysia, including Thailand, Laos, and Myanmar [3]. Cobras' venoms contain potent neurotoxic, cardiotoxic, and cytotoxic activities, attributed to protein families like three-finger toxins, cardiotoxins, and cytotoxins [4]. On the other hand, pit viper venoms can induce potent necrotic and hemotoxic effects through toxins such as L-amino acid oxidase and snake venom metalloproteinase, respectively [5].

The primary treatment for snake envenomation typically relies on administering horse-derived antisera, known as antivenom, to counteract snake venom's clinical and toxic effects [6]. While effective in preventing fatalities, the current antivenom presents several challenges. These include severe anaphylactic reactions and limited efficacy against snake venom from diverse geographical regions due to species-specific formulations [7]. Other factors effecting the efficacy of the antivenom includes the variation of venom caused by diet, sex and ontogenetic factor [8]. Moreover, the current formulation and treatment approach for antivenom has remained stagnant for many decades, highlighting the urgent need for a novel and innovative strategy to address snakebite envenomation. There are different approaches being studied, including small molecule inhibitor, peptide inhibitor and natural products where they target specific proteins [9]. Proteomics technique using tandem mass spectrometry has been the gold standard in profiling and characterising snake venom proteins. This approach enables scientists to accurately identify and profile different protein families within the crude venom of diverse snake species, facilitating the relative measurement of protein abundance and prediction of clinical outcomes following envenomation by these species [10,11]. Applying similar proteomic techniques, antigenic venom proteins from venomous snakes in other regions, such as India [12], South America [13], and African snakes [14], were successfully profiled. This comprehensive understanding of venom composition aids in the design and formulation of antivenom that is effective across various geographical regions and against different snake species, thereby enhancing the treatment outcomes for snakebite victims.

Therefore, in this present study, we investigated the antigenic venom proteins from five of the most common venomous snakes compassing of two families which are pit vipers, (*Calloselasma rhodostoma* and *Cryptelytrops purpureomaculatus*) and cobras (*Ophiophagus hannah*, *Naja kaouthia*, and *Naja sumatrana*). Using the agarose immunoprecipitation technique, we assayed the venom against various types of antivenom procured from Thailand (Queen Saovabha Memorial Institute, Bangkok). Subsequently, we employed high-resolution tandem mass spectrometry (LC-MS/MS) to identify the proteins from positive venom-antivenom interactions. These findings offer valuable insights that could contribute to the development of broad range antivenom.

## 2 Materials and methods

### 2.1 Crude venom collection

Crude venom of *Calloselasma rhodostoma* and *Cryptelytrops purpureomaculatus*) and cobras (*Ophiophagus hannah*, *Naja kaouthia*, and *Naja sumatrana* was purchased and collected from local and licensed venomous snake enthusiasts, Mr. Zainuddin Ismail (Bukit Bintang Enterprises Sdn. Bhd.) in Malaysia. The adult snakes are native to northern Malaysia (Perlis) and they are of both sex. For each species, the venom of 3–4 snakes were collected by gently placing the venom's fangs on a container wrapped with parafilm. The container was then sealed, stored in a cool box, and transported back to Monash Malaysia. Crude venom was then stored at −20 °C, followed by freeze-drying to preserve the venom activity. Freeze-dried crude venom was dissolved in double-distilled (Milli-Q) water before usage.

### 2.2 Antivenom

Antivenom was acquired from the Queen Saovabha Memorial Institute (QSMI) of the Thai Red Cross Society in Bangkok, Thailand, were used, i.e., king cobra antivenom (KCAV; LH00118), cobra antivenom (CAV; NK00117), Malayan pit viper antivenom (MPAV; NK00117), Russell's viper antivenom (RPAV; WR00308), hematopolyvalent antivenom (HPAV; HP00416), neuropolyvalent antivenom (NPAV; NP00116), banded krait antivenom (BKAV; BK00114) and Malayan krait antivenom (MKAV). The antivenom was diluted according to the information leaflet using the normal saline (0.9 % NaCl) provided.

### 2.3 Immunoprecipitation agarose gel assay

Agarose gel medium (2 g agarose in 100 mL MilliQ water) was pipetted on the glass slides and then allowed to polymerise. Two wells were made in the agarose gel with a distance of at least 1 cm between the wells and approximately 1 cm in diameter. Hundred microlitre of venom and antivenom were pipetted into one of each well and allowed to diffuse through the gel for 24–48 h. The venom-antivenom complex appeared as a white precipitate (white band) in the middle of the wells and proceeded to in-gel digestion and LC-MS/MS analysis.

### 2.4 In-gel tryptic digestion

After the excision of the gel band, an in-gel tryptic digestion protocol was conducted to digest the proteins on the band. The gel was prepared for digestion with 200 μL of a reduction buffer (3.1 mg of 10 mM dithiothreitol (DTT) in 2 mL of 50 mM ammonium bicarbonate) incubated at 56 °C for 60 min. The gel pieces were then centrifuged briefly before removing all liquid in the Eppendorf tube. Then, 200 μL of an alkylation buffer (20.4 mg of 55 mM iodoacetamide (IAM) in 2 mL of 50 mM ammonium bicarbonate) was added to the gel piece for incubation in the dark for 30 min, and all liquid was discarded after. The gel piece was then washed with 200 μL of 50 mM ammonium bicarbonate for 15 min before discarding all liquid and repeated with 200 μL of 50 mM ammonium bicarbonate in 50 % acetonitrile (ACN). The gel piece was washed with 200 μL of 100 % ACN for 15 min at 37 °C before being centrifuged briefly and all liquid contents discarded. 1 μL of Trypsin/Lys-C was added with 50 μL of 40 mM ammonium bicarbonate in 9 % ACN and left overnight at 37 °C. The digested sample was then centrifuged briefly, and its supernatant was collected in a separate collection tube. 50 μL of 5 % formic acid (FA) was added to the gel piece, vortexed briefly and incubated at 37 °C for 15 min. This process was repeated with 5 % FA in 50 % ACN and then with pure ACN. The supernatant was recovered into the collection tube at the end of each incubation. The gel piece was discarded, and the sample in the collection tube was subjected and spun to dry overnight in a vacuum concentrator before LC-MS/MS analysis.

### 2.5 LC-MS/MS analysis

The analysis was performed on Agilent 6550 Quadrupole Time-of-Flight (QTOF). Digested peptides (100 μg/ml) from in-solution tryptic digestion were loaded into an AdvancedBio Peptide Mapping, 2.1 × 250mm, 7 μm (pn 651750-902). Peptides were eluted with an increasing gradient, 5–100 % of 90 % acetonitrile in 0.1 % formic acid in water. The LCMS-QTOF parameters were set as positive polarity with the capillary voltage set at 2050 V and 300 V, respectively and 5 L/min of gas flow with a temperature of 300 °C. The spectrum was analysed in auto-MS mode, ranging from 110 to 3000 *m*/*z* for MS scan and 50–3000 *m*/*z* for MS/MS scan.

### 2.6 Data analysis using PEAKS Studio X plus

Antigenic proteins were identified and analysed using the UniProt database (Species: Serpentes and *Equus caballus*) through the PEAKS Studio X Plus software. The identification of the antigenic proteins was run against the Serpentes database, while the identification of the antibodies was run against the *Equus caballus* database. The profiling parameters include false discovery rate (FDR) at 0.1 %, −10logP values (>20), and a minimum of 1 unique protein identified for each result.

## 3 Results

### 3.1 Immunoprecipitation assay

**Fig. 1:**
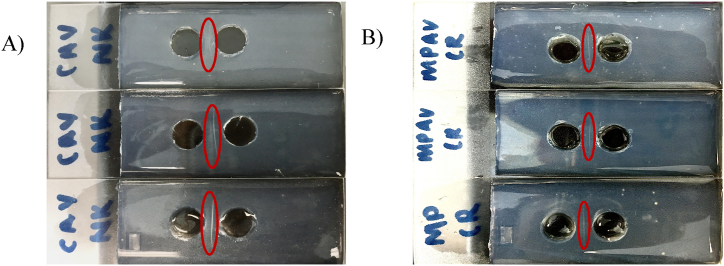
A). Positive interactions of the immunoprecipitation assay of cobra antivenom against *N. kaouthia*. B) Positive interactions of the immunoprecipitation assay of malayan pit viper antivenom against *C. rhodostoma*. The red circle highlights the precipitation between the venom and antivenom indication a positive interaction. (For interpretation of the references to colour in this figure legend, the reader is referred to the Web version of this article.)

The immunoprecipitation assay was conducted to investigate the interaction between venom and antivenom [king cobra antivenom (KCAV), cobra antivenom (CAV), Malayan pit viper antivenom (MPAV), Russell's viper antivenom (RPAV), hematopolyvalent antivenom (HPAV), neuropolyvalent antivenom (NPAV), banded krait antivenom (BKAV), and Malayan krait antivenom (MKAV)]. Positive interaction was indicated by the formation of white precipitate between the wells. Fig. 1A shows the positive interactions of the immunoprecipitation assay of Malayan pit viper antivenom against *C. rhodostoma* (Malayan pit viper) while Fig. 1B shows the positive interactions of the immunoprecipitation assay of cobra antivenom against *N. kaouthia (*Monocled cobra). Bands extracted from the immunoprecipitation assay underwent LC-MS/MS analysis to identify antigenic venom proteins. These proteins were then analysed against the UniProt database (Species: Serpentes) using PEAKS Studio X Plus software. Confirmation of venom protein binding with antivenoms was achieved by analysing against the *Equus caballus* database (Refer to supplementary documents).

The results of the immunoprecipitation assay were summarised in Table 1. Both *Naja* species showed same interactions with all the eight antivenom while *O. hannah* exhibited 5 positive interactions. Both the pit vipers presented with the same interactions across all the antivenom used.

**Table 1:**
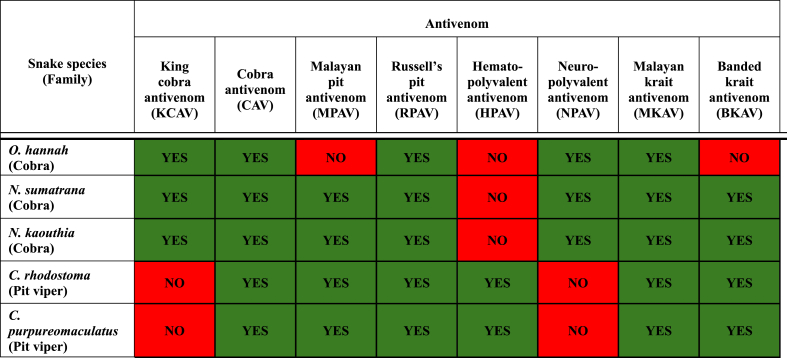
*Results of immunoprecipitation assay for all 5 crude venom against 8 antivenom*. The green highlighted boxes indicate positive interaction, and the red highlighted boxes indicates a negative interaction between the venom and antivenom. King cobra antivenom (KCAV), Cobra antivenom (CAV), Malayan pit antivenom (MPAV), Russell's pit antivenom (RPAV), Hemato-polyvalent antivenom (HPAV), Neuro-polyvalent antivenom (NPAV), Malayan krait antivenom (MKAV), Banded krait antivenom (BKAV).

### 3.2 *O. hannah* antigenic proteins

Table 2 summarised all the proteins identified from the immunoprecipitation assay from *Ophiophagus hannah*. Five proteins were identified in the interaction between *O. hannah* venom and KCAV: cysteine-rich venom proteins (CRVP), L-amino-acid oxidase (LAAO), beta-cardiotoxin, ShKT domain-containing protein, and alpha-elapitoxin. In the case of RPAV, seven proteins were identified: LAAO, amine oxidase, ophiophagus venom factor, SVMP-disintegrin, CRVP, ShKT domain-containing protein, and beta-cardiotoxin. Interaction with NPAV led to the identification of five proteins: LAAO, ophiophagus venom factor, SVMP-disintegrin, CRVP, and beta-cardiotoxin. Finally, interaction with MKAV revealed five proteins: CRVP, amine oxidase, LAAO, ShKT domain-containing protein, and beta-cardiotoxin. CRVP, LAAO, and amine oxidase were common proteins identified in interactions with all five antivenoms, while beta-cardiotoxin was present in all interactions except with CAV. Alpha-elapitoxin was only detected in interactions with elapid antivenom. Beta-cardiotoxin and cysteine-rich venom proteins exhibited the highest coverage among the identified proteins. Each protein contained at least one unique peptide in its sequence, with all −10logP values exceeding 40. All identified proteins were specific to the *O. hannah* species.

**Table 2:** *Proteins identified via LC-MS/MS from the O. hannah crude venom against KCAV, CAV, RPAV, NPAV. MKAV, and BKAV*. King cobra antivenom (KCAV), Cobra antivenom (CAV), Neuro-polyvalent antivenom (NPAV), Malayan krait antivenom (MKAV).

Accession	−10lgP	Coverage (%)	Unique	Avg. Mass	Description *(OS)*
*O. hannah* crude venom against KCAV					
Q7ZT98	197.6	31	10	26869	Cysteine-rich venom protein ophanin ***(O. hannah)***
P81383	152.54	21	5	55977	L-amino-acid oxidase ***(O. hannah)***
Q2VBN8	85.13	27	3	9380	Beta-cardiotoxin CTX9 ***(O. hannah)***
V8N8B4	163.58	12	1	24779	ShKT domain-containing protein ***(O. hannah)***
Q53B58	57.06	20	1	10210	Alpha-elapitoxin-Oh3a ***(O. hannah)***
***O. hannah* crude venom against CAV**					
P81383	215.81	40	5	55977	L-amino-acid oxidase ***(O. hannah)***
V8N3Q9	207.35	43	2	55593	Amine oxidase (Fragment) ***(O. hannah)***
I2C090	191.18	18	17	183927	Ophiophagus venom factor ***(O. hannah)***
A3R0T9	189.13	28	6	69049	Zinc metalloproteinase-disintegrin-like ohanin ***(O. hannah)***
Q7ZT98	185.82	51	14	26869	Cysteine-rich venom protein ophanin ***(O. hannah)***
Q53B58	174.67	59	7	10210	Alpha-elapitoxin-Oh3a ***(O. hannah)***
***O. hannah* crude venom against RPAV**					
P81383	243.32	43	3	55977	L-amino-acid oxidase ***(O. hannah)***
V8N3Q9	238.19	46	3	55593	Amine oxidase (Fragment) ***(O. hannah)***
I2C090	206.75	15	11	183927	Ophiophagus venom factor ***(O. hannah)***
A3R0T9	197.39	25	14	69049	Zinc metalloproteinase-disintegrin-like ohanin ***(O. hannah)***
Q7ZT98	180.33	55	17	26869	Cysteine-rich venom protein ophanin ***(O. hannah)***
tV8N8B4	170.3	48	2	24779	ShKT domain-containing protein ***(O. hannah)***
Q53B46	110.47	61	6	9352	Beta-cardiotoxin CTX15 ***(O. hannah)***
***O. hannah* crude venom against NPAV**					
I2C090	182.32	21	13	183927	Ophiophagus venom factor ***(O. hannah)***
Q7ZT98	158.23	46	4	26869	Cysteine-rich venom protein ophanin ***(O. hannah)***
A3R0T9	139.75	61	12	69049	Zinc metalloproteinase-disintegrin-like ohanin ***(O. hannah)***
P81383	135.33	32	15	55977	L-amino-acid oxidase ***(O. hannah)***
Q53B46	104.21	61	6	9352	Beta-cardiotoxin CTX15 ***(O. hannah)***
***O. hannah* crude venom against MKAV**					
Q7ZT98	199.17	33	5	26869	Cysteine-rich venom protein ophanin ***(O. hannah)***
V8N3Q9	146.06	14	6	55593	Amine oxidase (Fragment) ***(O. hannah)***
P81383	146.06	14	6	55977	L-amino-acid oxidase ***(O. hannah)***
V8N8B4	188.08	16	1	24779	ShKT domain-containing protein ***(O. hannah)***
Q2VBN8	45.61	27	2	9380	Beta-cardiotoxin CTX9 ***(O. hannah)***

### 3.3 *N. sumatrana* antigenic proteins

Table 3 summarised the proteins identified from all interactions involving *Naja sumatrana*. Predominantly, cytotoxins were identified as the common antigenic proteins across interactions with seven antivenoms. These include cytotoxin isoforms 4b, 4a, 3a, KJC3, 2, 1, and 3. Notably, cytotoxin KJC3 and cytotoxin 4b from *Naja sputatrix* were consistently identified across all interactions. In the interaction with KCAV, seven proteins were identified: cytotoxin 4b, 4a, 3a, KJC3, 2, 1, and 3. Similarly, six proteins were determined from the interaction with CAV, including cytotoxin KJC3, 4b, 4a, 5, and 2, along with neutral phospholipase A2. MPAV interaction revealed cytotoxin KJC3, 4b, 4a, 1, and 2, alongside weak neurotoxin 6 and weak neurotoxin 8. RPAV interaction identified cytotoxin KJC3, 4b, 3a, 4a, and 1, along with neutral phospholipase A2. Interaction with NPAV unveiled cytotoxin KJC3, 4b, 4a, 1, and 3a, along with weak neurotoxin 6. BKAV interaction resulted in the identification of five proteins: cytotoxin 4b, 3a, KJC3, 2, and 7. Lastly, MKAV interaction identified cytotoxin 4b, KJC3, 2, 1, and 3. All proteins contain at least one unique peptide sequence, with −10logP values exceeding 40. The proteins identified from the database belong to the *Naja* genus.

**Table 3:** *Proteins identified via LC-MS/MS from the N. sumatrana crude venom against KCAV, CAV, MPAV, RPAV, NPAV. MKAV, and BKAV*. King cobra antivenom (KCAV), Cobra antivenom (CAV), Malayan pit antivenom (MPAV), Russell's pit antivenom (RPAV), Neuro-polyvalent antivenom (NPAV), Malayan krait antivenom (MKAV), Banded krait antivenom (BKAV).

Accession	−10lgP	Coverage (%)	Unique	Avg. Mass	Description *(OS)*
***N. sumatrana* crude venom against KCAV**					
O73856	211.24	74	2	9084	Cytotoxin 4b ***(N. sputatrix)***
Q98959	201.16	74	1	9065	Cytotoxin 3a ***(N. atra)***
O93473	209.07	74	1	9068	Cytotoxin 4a **(*N. sputatrix)***
P60311	203.85	100	3	6753	Cytotoxin KJC3 **(*N. sputatrix)***
P01440	196.07	100	2	6763	Cytotoxin 2 ***(N. naja)***
P0CH80	195.54	87	1	6807	Cytotoxin 1 ***(N. kaouthia)***
P01459	162.87	70	1	6839	Cytotoxin 3 ***(N. annulifera)***
***N. sumatrana* crude venom against CAV**					
P60311	213.52	100	5	6753	Cytotoxin KJC3 **(*N. sputatrix)***
O73856	206.02	74	2	9084	Cytotoxin 4b **(*N. sputatrix)***
O93473	203.82	74	2	9068	Cytotoxin 4a **(*N. sputatrix)***
P24779	143.27	92	1	6654	Cytotoxin 5 ***(N. kaouthia)***
P01440	221.91	100	1	6763	Cytotoxin 2 ***(N. naja)***
Q92084	147.73	44	7	16189	Neutral phospholipase A2 muscarinic inhibitor **(*N. sputatrix)***
***N. sumatrana* crude venom against MPAV**					
O73856	211.08	74	2	9084	Cytotoxin 4b **(*N. sputatrix)***
O93473	208.52	74	2	9068	Cytotoxin 4a **(*N. sputatrix)***
P60311	206.01	100	4	6753	Cytotoxin KJC3 **(*N. sputatrix)***
P01440	212.59	100	1	6763	Cytotoxin 2 ***(N. naja)***
P0CH80	207.96	100	2	6807	Cytotoxin 1 ***(N. kaouthia)***
O42256	205.16	71	3	9807	Weak neurotoxin 6 **(*N. sputatrix)***
Q802B3	204.9	71	1	9809	Weak neurotoxin 8 **(*N. sputatrix)***
***N. sumatrana* crude venom against RPAV**					
A0A7T7DMY7	199.41	74	2	9054	Cytotoxin 1 ***(N. sumatrana)***
O73856	194.33	69	1	9084	Cytotoxin 4b **(*N. sputatrix)***
P60311	192.51	97	1	6753	Cytotoxin KJC3 **(*N. sputatrix)***
Q98959	200.03	74	2	9065	Cytotoxin 3a ***(N. atra)***
O93473	187.72	74	1	9068	Cytotoxin 4a **(*N. sputatrix)***
Q92084	142.3	50	6	16189	Neutral phospholipase A2 muscarinic inhibitor **(*N. sputatrix)***
***N. sumatrana* crude venom against NPAV**					
P60311	204.07	88	2	6753	Cytotoxin KJC3 **(*N. sputatrix)***
O73856	210.07	72	1	9084	Cytotoxin 4b **(*N. sputatrix)***
O93473	208.53	77	2	9068	Cytotoxin 4a **(*N. sputatrix)***
P0CH80	161.65	82	1	6807	Cytotoxin 1 ***(N. kaouthia)***
Q98959	229.39	77	2	9065	Cytotoxin 3a (***N. atra)***
O42256	190.64	66	12	9807	Weak neurotoxin 6 **(*N. sputatrix)***
***N. sumatrana* crude venom against MKAV**					
A0A7T7DMY7	221.01	74	4	9054	Cytotoxin 1 (***N. sumatrana)***
O73856	217.17	74	1	9084	Cytotoxin 4b (***N. sputatrix)***
P60311	208.21	90	2	6753	Cytotoxin KJC3 (***N. sputatrix)***
P01459	159.08	75	3	6839	Cytotoxin 3 (***N. annulifera)***
P01440	188.22	100	1	6763	Cytotoxin 2 (***N. naja)***
***N. sumatrana* crude venom against BKAV**					
O73856	266.94	74	1	9084	Cytotoxin 4b ***(N. sputatrix)***
P60311	251.44	100	3	6753	Cytotoxin KJC3 ***(N. sputatrix)***
P01440	247.06	100	1	6763	Cytotoxin 2 ***(N. naja)***
Q98959	222.82	74	1	9065	Cytotoxin 3a ***(N. atra)***
O73859	267.43	97	1	7062	Cytotoxin 7 (Fragment) ***(N. sputatrix)***

### 3.4 *N. kaouthia* antigenic proteins

The proteins identified from all *N. kaouthia* interactions were summarised in Table 4. In the interaction with KCAV, the identified proteins include cytotoxin 3, cytotoxin 2, cytotoxin 2a, cytotoxin 1d/1e, and cytotoxin 2c. Interaction with CAV revealed cytotoxin KJC3, cytotoxin 3, cytotoxin 10, cytotoxin 1d/1e, and cytotoxin 1. MPAV interaction resulted in the identification of cytotoxin 5, cytotoxin 2, cytotoxin 1d/1e, cytotoxin 1a, and tryptophan-containing weak neurotoxin. RPAV interaction led to the identification of cytotoxin KJC3, cytotoxin 3, cytotoxin 4a, cytotoxin 1d/1e, and cytotoxin 1a. Interaction with NPAV identified cytotoxin 1d/1e, cytotoxin 2, venom phosphodiesterase, and CRVP. MKAV interaction identified cytotoxin 3, cytotoxin 10, cytotoxin 1d/1e, cytotoxin 1, and acidic phospholipase A2. Lastly, interaction with BKAV resulted in the identification of cytotoxin VC-1, cytotoxin 3, cytotoxin 1d/1e, cytotoxin 10, and tryptophan-containing weak neurotoxin. All identified proteins contain at least one unique peptide sequence, with −10logP values exceeding 40. These proteins belong to the *Naja* genus as identified from the sample using the database.

**Table 4:** *Proteins identified via LC-MS/MS from N. kaouthia crude venom against KCAV, CAV, MPAV, RPAV, NPAV, MKAV and BKAV.* King cobra antivenom (KCAV), Cobra antivenom (CAV), Malayan pit antivenom (MPAV), Russell's pit antivenom (RPAV), Neuro-polyvalent antivenom (NPAV), Malayan krait antivenom (MKAV), Banded krait antivenom (BKAV).

Accession	−10lgP	Coverage (%)	Unique	Avg. Mass	Description *(OS)*
***N. kaouthia* crude venom against KCAV**					
P01446	162.15	100	3	6717	Cytotoxin 3 ***(N. kaouthia)***
P01440	153.53	100	1	6763	Cytotoxin 2 ***(N. naja)***
P86538	115.11	77	1	6711	Cytotoxin 2a ***(N. naja)***
Q98958	180.79	73	2	8992	Cytotoxin 1d/1e ***(N. atra)***
O93472	153.56	74	1	9128	Cytotoxin 2c ***(N. sputatrix)***
***N. kaouthia* crude venom against CAV**					
Q98958	226.56	72	3	8992	Cytotoxin 1d/1e ***(N. atra)***
P60311	204.82	98	1	6753	Cytotoxin KJC3 ***(N. sputatrix)***
P86541	217.5	100	1	6764	Cytotoxin 10 ***(N. naja)***
P01446	236.57	100	2	6717	Cytotoxin 3 ***(N. kaouthia)***
P01451	191.98	92	1	6821	Cytotoxin 1 ***(N. oxiana)***
***N. kaouthia* crude venom against MPAV**					
Q98958	200.51	73	3	8992	Cytotoxin 1d/1e ***(N. atra)***
Q98957	190.66	72	1	8976	Cytotoxin 1a ***(N. atra)***
P07525	170.94	87	1	6810	Cytotoxin 5 ***(N. atra)***
P01440	163.56	80	1	6763	Cytotoxin 2 ***(N. naja)***
P82935	200.59	67	15	9915	Tryptophan-containing weak neurotoxin ***(N. kaouthia)***
***N. kaouthia* crude venom against RPAV**					
Q98958	173.3	65	1	8992	Cytotoxin 1d/1e ***(N. atra)***
P01446	142.21	87	2	6717	Cytotoxin 3 ***(N. kaouthia)***
P60311	145.61	78	1	6753	Cytotoxin KJC3 ***(N. sputatrix)***
Q98957	164.24	58	1	8976	Cytotoxin 1a ***(N. atra)***
O93473	137.84	58	1	9068	Cytotoxin 4a ***(N. sputatrix)***
***N. kaouthia* crude venom against NPAV**					
A0A2D0TC04	183.48	23	17	94616	Venom phosphodiesterase ***(N. atra)***
P84805	160.04	29	3	26846	Cysteine-rich venom protein kaouthin-1 ***(N. kaouthia)***
P01445	159.76	87	1	6745	Cytotoxin 2 ***(N. kaouthia)***
Q98958	161.89	64	1	8992	Cytotoxin 1d/1e ***(N. atra)***
***N. kaouthia* crude venom against MKAV**					
Q98958	157.76	65	1	8992	Cytotoxin 1d/1e ***(N. atra)***
P01446	163	97	3	6717	Cytotoxin 3 ***(N. kaouthia)***
P01451	171.51	82	1	6821	Cytotoxin 1 ***(N. oxiana)***
P00597	172.32	69	1	16016	Acidic phospholipase A2 (***N. kaouthia)***
P86541	134.67	62	1	6764	Cytotoxin 10 ***(N. naja)***
***N. kaouthia* crude venom against BKAV**					
Q98958	184.06	72	1	8992	Cytotoxin 1d/1e ***(N. atra)***
Q9PS33	186.73	92	1	6724	VC-1 cytotoxin ***(N. oxiana)***
P01446	171.49	97	4	6717	Cytotoxin 3 ***(N. kaouthia)***
P86541	148.45	80	2	6764	Cytotoxin 10 ***(N. naja)***
P82935	169.17	66	9	9915	Tryptophan-containing weak neurotoxin ***(N. kaouthia)***

### 3.5 *C. rhodostoma* antigenic proteins

The proteins identified from all *C. rhodostoma* interactions were summarised in Table 5. The common antigenic proteins identified across all interactions of *C. rhodostoma* venom against six antivenoms were PLA_2_. Snaclec rhodocetin subunit beta was consistently identified in every interaction except with CAV, while snaclec rhodocetin subunit alpha was present in almost every interaction except with MKAV and BKAV. Zinc metalloproteinase/disintegrin was identified in all interactions except against HPAV. Snake venom metalloproteinase kistomin was identified in interactions with CAV, HPAV, MKAV, and BKAV. Lastly, LAAO was detected in the interaction with MPAV, HPAV, MKAV, and BKAV. All identified proteins contain at least one unique peptide sequence, with −10logP values exceeding 40. These proteins were matched with the *Calloselasma rhodostoma* species in the database.

**Table 5:** *Proteins identified via LC-MS/MS from C. rhodostoma crude venom against CAV, MPAV, RPAV, HPAV, MKAV and BKAV*. Cobra antivenom (CAV), Malayan pit antivenom (MPAV), Russell's pit antivenom (RPAV), Hemato-polyvalent antivenom (HPAV), Malayan krait antivenom (MKAV), Banded krait antivenom (BKAV).

Accession	−10lgP	Coverage (%)	Unique	Avg. Mass	Description *(OS)*
***C. rhodostoma* crude venom against CAV**					
P30403	185.86	17	14	54006	Zinc metalloproteinase/disintegrin ***(C. rhodostoma)***
P81398	160.51	59	15	15190	Snaclec rhodocetin subunit beta ***(C. rhodostoma)***
A0A0H3U266	160.8	68	5	15486	Phospholipase A2 ***(C. rhodostoma)***
P0CB14	133.27	20	7	47446	Snake venom metalloproteinase kistomin ***(C. rhodostoma)***
***C. rhodostoma* crude venom against MPAV**					
P81397	137.25	83	8	15962	Snaclec rhodocetin subunit alpha ***(C. rhodostoma)***
P81382	166.86	46	16	58221	L-amino-acid oxidase ***(C. rhodostoma)***
P81398	150.91	59	10	15190	Snaclec rhodocetin subunit beta ***(C. rhodostoma)***
A0A0H3U266	150	58	5	15486	Phospholipase A2 ***(C. rhodostoma)***
P30403	181.83	22	11	54006	Zinc metalloproteinase/disintegrin ***(C. rhodostoma)***
***C. rhodostoma* crude venom against RPAV**					
P81397	182.81	80	9	15962	Snaclec rhodocetin subunit alpha ***(C. rhodostoma)***
P81398	171.56	59	14	15190	Snaclec rhodocetin subunit beta ***(C. rhodostoma)***
A0A0H3U266	149.25	43	4	15486	Phospholipase A2 ***(C. rhodostoma)***
Q9PVF4	143.51	42	2	15457	Basic phospholipase A2 homolog W6D49 ***(C. rhodostoma)***
P30403	136.12	10	7	54006	Zinc metalloproteinase/disintegrin ***(C. rhodostoma)***
***C. rhodostoma* crude venom against HPAV**					
P81382	219	70	25	58221	L-amino-acid oxidase ***(C. rhodostoma)***
P81398	159.43	70	12	15190	Snaclec rhodocetin subunit beta ***(C. rhodostoma)***
A0A0H3U266	138.8	43	11	15486	Phospholipase A2 ***(C. rhodostoma)***
P81397	179.33	80	11	15962	Snaclec rhodocetin subunit alpha ***(C. rhodostoma)***
P0CB14	218.61	37	24	47446	Snake venom metalloproteinase kistomin ***(C. rhodostoma)***
***C. rhodostoma* crude venom against MKAV**					
P81382	181.91	60	19	58221	L-amino-acid oxidase ***(C. rhodostoma)***
P81398	135.22	58	8	15190	Snaclec rhodocetin subunit beta ***(C. rhodostoma)***
P30403	134.28	13	5	54006	Zinc metalloproteinase/disintegrin ***(C. rhodostoma)***
A0A0H3U266	132.5	43	4	15486	Phospholipase A2 ***(C. rhodostoma)***
P0CB14	127.08	21	10	47446	Snake venom metalloproteinase kistomin ***(C. rhodostoma)***
Q9PVF4	124.81	42	2	15457	Basic phospholipase A2 homolog W6D49 ***(C. rhodostoma)***
***C. rhodostoma* crude venom against BKAV**					
A0A0H3U266	204.27	52	3	15486	Phospholipase A2 ***(C. rhodostoma)***
P81398	199.58	59	7	15190	Snaclec rhodocetin subunit beta ***(C. rhodostoma)***
P30403	149.41	13	5	54006	Zinc metalloproteinase/disintegrin ***(C. rhodostoma)***
P0CB14	148.7	15	9	47446	Snake venom metalloproteinase kistomin ***(C. rhodostoma)***
P81382	134.69	27	8	58221	L-amino-acid oxidase ***(C. rhodostoma)***

### 3.6 *C. purpureomaculatus* antigenic proteins

The proteins identified from all *C. purpureomaculatus* interactions were summarised in Table 6. PLA_2_ was identified as the common antigenic protein across all interactions of *C. purpureomaculatus* venom with six antivenoms. Snaclec rhodocetin subunit beta was consistently identified in every interaction except with RPAV and BKAV, while snaclec rhodocetin subunit alpha was present in three interactions: CAV, MPAV, and MKAV. Snaclec coagulation factor was detected in the interaction of the crude venom with CAV, MPAV, and RPAV. LAAO was only identified in interactions with MKAV and HPAV. Zinc metalloproteinase/disintegrin was solely identified in interactions with HPAV. All identified proteins contain at least one unique peptide sequence, with −10logP values exceeding 40. These proteins were matched with the *Cryptelytrops* species, a subset of the *Trimeresurus* family, in the database.

**Table 6:** *Proteins identified via LC-MS/MS from the C. purpureomaculatus crude venom against, CAV, MPAV, RPAV, HPAV. MKAV, and BKAV.* Cobra antivenom (CAV), Malayan pit antivenom (MPAV), Russells pit antivenom (RPAV), Hemato-polyvalent antivenom (HPAV), Malayan krait antivenom (MKAV), Banded krait antivenom (BKAV).

Accession	−10lgP	Coverage (%)	Unique	Avg. Mass	Description *(OS)*
***C. purpureomaculatus* crude venom against CAV**					
P0DJL3	155.05	85	9	14498	Snaclec purpureotin subunit beta ***(T. purpureomaculatus)***
A0A0H3U1W4	137.69	57	1	15895	Phospholipase A2 ***(T. albolabris)***
Q71RR4	131.02	42	4	17092	Snaclec coagulation factor IX/factor X-binding protein subunit A ***(T. stejnegeri)***
***C. purpureomaculatus* crude venom against MPAV**					
A0A0H3U245	166.9	51	3	15197	Phospholipase ***(T. albolabris)***
P0DJL3	129.87	53	7	14498	Snaclec purpureotin subunit beta ***(T. purpureomaculatus)***
Q71RR4	115.37	43	5	17092	Snaclec coagulation factor IX/factor X-binding protein subunit A ***(T. stejnegeri)***
P0DJL2	102.04	52	3	15613	Snaclec purpureotin subunit alpha ***(T. purpureomaculatus)***
***C. purpureomaculatus* crude venom against RPAV**					
A0A0H3U1W4	141.04	51	1	15895	Phospholipase A2 ***(T. albolabris)***
Q71RR4	146.93	55	6	17092	Snaclec coagulation factor IX/factor X-binding protein subunit A ***(T. stejnegeri)***
***C. purpureomaculatus* crude venom against HPAV**					
A0A0H3U267	132.93	36	5	15239	Phospholipase A2 ***(T. albolabris)***
P0DJL3	128.15	60	1	14498	Snaclec purpureotin subunit beta ***(T. purpureomaculatus)***
Q6WP39	120.49	15	2	58601	L-amino-acid oxidase ***(T. stejnegeri)***
P0C6E8	121.69	14	9	48204	Zinc metalloproteinase/disintegrin (Fragment) ***(T. gramineus)***
***C. purpureomaculatus* crude venom against MKAV**					
A0A0H3U245	161.1	59	3	15197	Phospholipase A2 ***(T. albolabris)***
P0DJL3	112.96	37	5	14498	Snaclec purpureotin subunit beta ***(T. purpureomaculatus)***
Q6WP39	111.82	14	1	58601	L-amino-acid oxidase ***(T. stejnegeri)***
P0DJL2	118.9	52	5	15613	Snaclec purpureotin subunit alpha ***(T. purpureomaculatus)***
***C. purpureomaculatus* crude venom against BKAV**					
A0A0H3U239	133.98	17	3	15816	Phospholipase A2 ***(T. erythrurus)***

## 4 Discussion

The current study focuses on the identification of antigenic proteins from five medically significant snakes in Malaysia using immunoprecipitation assay and LC-MS/MS. It is essential to analyse the venom composition to further understand the effects of envenomation and how can the treatment of snakebite be improved. The current antivenom that are available are either species specific (monovalent: raised against one crude venom) or syndromic antivenom (polyvalent: raised against snake venom with similar envenomation symptoms). Studying the venom composition allows us to identify and categorise them into the correct family and genus while also providing insights into the evolutionary history and adaptation of the snakes [15]. In this case, the information gathered from venom analysis was used to identify the antigenic proteins that are present in the five medically significant Malaysian venomous snakes (*Ophiophagus hannah, Naja sumatrana, Naja kaouthia, Calloselasma rhodostoma,* and *Cryptelytrops purpureomaculatus*). The immunoprecipitation assay is a technique utilised to study protein-protein interactions, based on the principle of antibody binding to the protein of interest. It involves the use of a medium, such as agarose, through which proteins and antibodies can diffuse. This facilitates the binding of both proteins and antibodies to form a precipitate, typically observed as a white band [16]. This approach enables the detection of protein binding, as well as the screening and purification of the protein complex that forms the band [16]. One of the main advantages of this approach is its simplicity and cost-effectiveness. Moreover, it is reproducible, as demonstrated by its repeated use with various venom and antivenom combinations in this study.

After observing the interaction on the agarose assay, LC-MS/MS was employed to identify the proteins bound to the antibodies from the antivenom. This approach was chosen due to its superior sensitivity compared to other methods such as gel electrophoresis or enzyme-linked immunosorbent assay (ELISA) [17]. LC-MS/MS operates with high-speed, high-throughput, and automated processing, facilitating the deep detection of trace protein components [18,19], crucial for identifying all proteins digested from the gel. Numerous studies have utilised the LC-MS/MS approach to profile snake venoms and determine the relative abundance of protein compositions [10,20]. For example, proteins from two pit vipers, *Tropidolaemus wagleri* and *Cryptelytrops purpureomaculatus,* were identified, and relative abundance of the venom proteins was detected using LC-MS/MS [10]. Prior to that, the venom proteome of *Naja haje*, a cobra species, was documented using chromatographic fractionation and Nano ESI-liquid chromatography and tandem mass spectrometry (nano-ESI-LCMS/MS) [18].

### 4.1 Crude venom and antivenom cross reactivity

The white band (Fig. 1) formed in the immunoprecipitation assay confirms there are interactions between the protein from the venom and the antibodies from the antivenom. The formation of the white band in the immunoprecipitation assay represents visual confirmation of interactions between the venom protein and the antibodies from the antivenom [21]. This assay demonstrates antivenom cross-reactivity, where the antivenom recognises a linear epitope on the protein originating from multiple snake species. A linear epitopic element may cover either the entire epitope sequence or a partial sequence that potentially folds into a structure mimicking the conformational epitope, fitting the paratopes of the antibodies. In polyclonal antivenom development, cross-reactivity refers to the antivenom's ability to effectively bind with antigens from various snake species, achieved through immunisation with venoms from diverse snake species [22].

Proposed theories of cross-reactivity suggest that monoclonal antibodies may possess cross-binding abilities toward multiple proteins due to antibody tolerance regarding antigen variation [23] or that polyclonal antibodies constitute a pool of monoclonal antibodies capable of binding to all toxins present in the venom [22]. While other studies on cross-reactivity have been conducted [[24], [25], [26]], the precise mechanism contributing to these observations remains unknown. Despite the undetermined mechanism, the concept of cross-reactivity holds potential benefits for antivenom production.

Among the eight antivenoms used in this study, six of them are monovalent (monospecific) antivenom; KCAV, CAV, MPAV, RPAV, MKAV, and BKAV, while the remaining two are polyvalent (polyspecific) antivenoms; NPAV and HPAV. Monospecific antivenoms target venom from a single species, whereas polyvalent antivenoms are effective against multiple species [27,28]. CAV, RPAV, and MKAV exhibited positive interactions across all venoms, potentially driven by a short motif of conserved residues present across the identified proteins of all five venoms, regardless of their families [29]. KCAV showed positive interaction with venoms from *O. hannah, N. sumatrana,* and *N. kaouthia*. The monospecific antibodies in KCAV are specific to proteins in *O. hannah,* as it was raised against its crude venom. Interestingly, KCAV also demonstrated positive interactions with two other vipers, due to the presence of heterologous proteins with similar antigenic properties as the venom used during immunisation [30]. These heterologous venom proteins may fold to fit the structure of the paratope, leading to neutralisation by the antibodies [21].

MPAV and BKAV exhibited positive interactions for the immunoprecipitation assay, except with *O. hannah* crude venom. Although these antivenoms did show positive interactions with the other two cobras, *N. sumatrana* and *N. kaouthia,* they failed to form a white precipitate with *O. hannah*. These absence of may be due to the lack of epitope recognition by the antibodies, as *Ophiophagus hannah* is not considered a "true cobra" [31]. Even though O*. hannah* comes under the cobra family, they may not possess similar proteins to the other two cobras in this study. Hence why MKAV and BKAV showed positive interactions with both the cobras but not *O. hannah*. Due to evident taxonomic differences, they possess distinct venom proteins leading to differing results from those observed with the other two cobras. King cobra anatomically differs from members of the *Naja* genus and is more closely related to the mambas [32]. It is expected that KCAV would exhibit a positive interaction with all the cobras as it was raised against the king cobra crude venom.

NPAV and HPAV, are syndromic polyvalent antivenom designed to address snake envenomation symptoms manifested by the victims [28,33]. NPAV comprises immunogens from *Naja kaouthia, Ophiophagus hannah,* and *Bungarus fasiatus* [34] and *Bungarus candidus*. NPAV demonstrated a positive interaction with all three elapids and negative interaction with the vipers. NPAV targets envenomation by elapids, whose venom primarily consists of neurotoxins and cytotoxins, aligning with the assay results from our present study [28,34]. The antibodies in NPAV can bind to the heterologous antigenic venom proteins in the cobras [28]. Due to the nature of the NPAV and the venom from elapids, the findings obtained from the immunoprecipitation assay corresponds to the function of NPAV.

HPAV, produced using venoms from *Daboia russellii, Calloselasma rhodostoma*, and *Trimeresurus albolabris*, targets hematologic disorders typically presented in viper envenomation victims, characterised by incoagulable blood and prolonged clotting time [35]. This is due to the composition of viper venom that are predominantly hemotoxic. The HPAV interaction from our present study showed positive reaction with both the vipers used in this study and the positive reactions are anticipated, as *C. rhodostoma* venom was used in its production. Since *C. purpureomaculatus* was formerly classified as *Trimeresurus purpureomaculatus*, it shares similar proteins with the crude venom used to produce hematopolyvalent antivenom. The proteins in *C. purpureomaculatus* have much similar to proteins found within the *Trimeresurus* family venom such as SVMP and PLA_2_ [10]. While cross-reactivity between venoms and antivenoms was observed in our present study, identifying the proteins responsible for these interactions is crucial for venomics research. This information could be instrumental in fortifying plasma-derived antivenoms with monoclonal antibodies, potentially broadening neutralisation capacity or enhancing efficacy against key toxins. Improving antivenom specificity can reduce adverse effects and have broader applicability across different demographics.

### 4.2 Antigenic protein across cobra and pit viper venom

Snake envenomation leads to various pathological effects, including neurotoxicity, hemotoxicity, and cytotoxicity at the bite site [36]. These diverse clinical manifestations are due to the variations in the toxin constituents within the venom. Our LC-MS/MS analysis of the protein bands from the immunoprecipitation assay demonstrated antigenic proteins specific to certain snake families and species (see Table 7). We identified eight antigenic protein families across the five species studied. However, distinct differences were identified in the antigenic proteins found in cobras and pit vipers alongside shared proteins. 3FTx (neurotoxins) and CRVP are antigenic in elapids, reflecting the prominent neurotoxic effects of cobra envenomation [37]. Although elapids exhibit similar symptoms, CRVP was only found to be antigenic in king cobra interactions. Conversely, proteins specific to pit vipers, such as Snaclec and SVMP (P-I and P-II class), were identified. However, despite varying post-envenomation effects, PLA_2_ and LAAO emerge as common antigenic proteins in both cobras and pit vipers. Despite the similarity in names, venom proteins can exhibit diverse immunogenicity, affecting their ability to bind to antibodies. Hence, identifying the antigenic properties in venom holds promise for developing effective, broad-spectrum antivenoms.

**Table 7:**
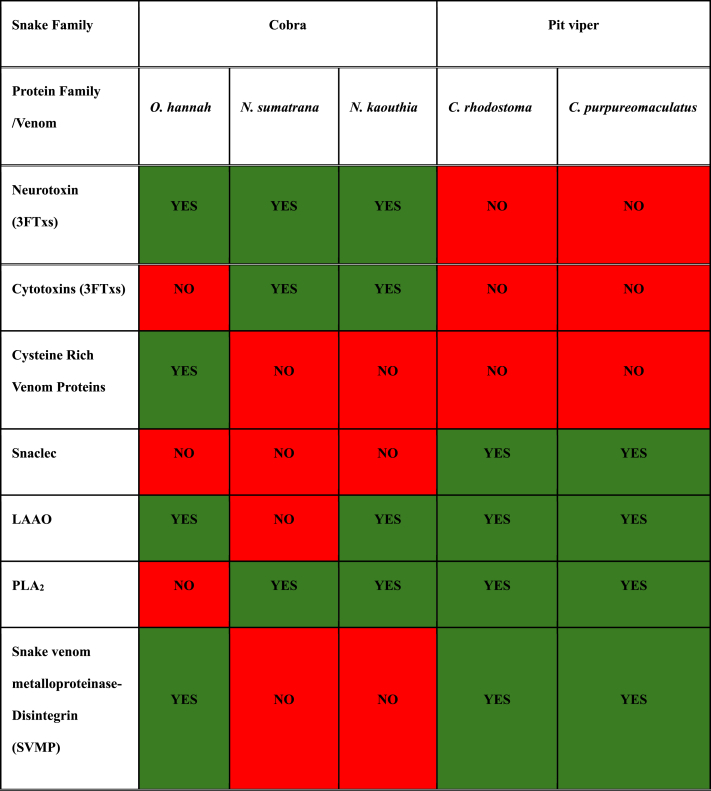
*Common antigenic proteins across all venom and antivenom interactions*. The green highlighted boxes indicate presence of the antigenic protein, and the red highlighted boxes indicate an absence of the antigenic protein from the interactions. King cobra antivenom (KCAV), Cobra antivenom (CAV), Malayan pit antivenom (MPAV), Russells pit antivenom (RPAV), Hemato-polyvalent antivenom (HPAV), Neuro-polyvalent antivenom (NPAV), Malayan krait antivenom (MKAV), Banded krait antivenom (BKAV).

### 4.3 Common antigenic proteins in cobra

Our study identified 3FTXs and cytotoxins as the common antigenic protein across the three cobras. The clinical manifestations of cobra envenomation include severe neurotoxic effects such as limb weakness, respiratory muscle paralysis and paraesthesia [38]. Major venom families found in king cobra include LAAO, metalloprotease, CRVP, PLA_2_ and 3FTxs (Chang, Tsai, and Tsai 2013). In the *Naja* genus, the key toxins are PLA_2_, neurotoxins, and cytotoxins/cardiotoxins (Yap et al., 2014). These cobra proteins are responsible for the above biological symptoms onto the victim.

#### 4.3.1 Three-finger toxins (3FTxs)

3FTxs comprises of three parallel β-sheets, with multiple disulphide bonds. The specific biological effects induced by 3FTxs heavily depend on the toxin subtype and the target receptor or ion channel [39] inducing neurotoxicity, cardiotoxicity anticoagulation and cytotoxicity [40]. Additionally, the interaction of 3FTx with the lipid bilayer of the cell leads to the disruption of the cell membrane and subsequent physiological changes in cell metabolism [41,42]. The three main types of 3FTX are short-chain, long-chain and nonconventional 3FTx [43]. On average, 3FTx constitutes approximately 51.3 % of the venom composition in elapids [39].

##### 4.3.1.1 Neurotoxin

There are two kinds of neurotoxins that are particularly harmful to the human body: A) α-neurotoxins, which acts as a non-enzymatic acetylcholine receptor (AchrRs) blockers and B) β-neurotoxins, which functions as a pre-synaptic phospholipase A2 [44]. α-neurotoxins, classified into three main groups-long chain, short chain, and non-conventional α-neurotoxins are post-synaptically active neurotoxins that bind to the post-synaptic muscle nAChRs. Generally, α-neurotoxins’ binding are irreversibly even when administering of antivenom or acetylcholinesterase inhibitors (AChEIs) [45]. Neurotoxin proteins in snake venom target the nervous system, leading to muscle paralysis, respiratory failure, and neurological symptoms [45].

##### 4.3.1.2 Cytotoxin

Various isoforms of cytotoxins were identified from the interaction of *Naja* venom against all the antivenom. Cytotoxins constitute approximately 40–70 % of *Naja* venom and they are highly amphipathic proteins. These cytotoxins mediate a range of biological processes, including depolarizing excitable membranes of heart cells and neurons, modulating the activity of membrane-bound enzymes, inhibiting platelet aggregation, inducing haemolytic and cytotoxic activity, and triggering a cardiac arrest [44,46,47]. The majority cytotoxin-mediated toxicity arises from their ability to bind to cell membranes, subsequently altering the structure and function of the lipid bilayer [44]. Cytotoxins act on various cell types, such as red blood cells, lymphocytes, tumour cells, spleen cells and cardiac myocytes. However, the pathological effects depend on the proteins found on the cell membrane and the phospholipids on the outer part of the plasma membrane. Generally, cytotoxins kill cells by disrupting the cell membranes [48]. The predominant antigenic proteins in the *Naja* venom are cytotoxins of various isoforms causing effects such as oedema, severe blistering, and necrosis. Furthermore, these effects can lead to secondary injuries including limb loss due to severe local tissue damage [44,49]. Targeting these proteins while formulating an antivenom may help prevent rapid swelling, enlargement of lymph nodes, or necrosis at the bite site.

##### 4.3.1.3 Β-cardiotoxin

β-cardiotoxin from *O. hannah* is a relatively new protein identified in 2007, exhibiting a distinct structure and function compared to other cardiotoxins [50]. Despite sharing similar sequences with conventional cytotoxins, β-cardiotoxin as an individual protein is less harmful than cytotoxins. β-cardiotoxin has been shown to decrease heart rate while acting by directly acting on cardiac tissue [50]. The combined action of various 3FTxs has been postulated to produce synergistic antagonising effects, leading to neuromuscular paralysis along respiratory failure in snakebite victims [51]. Inhibitors of 3FTx include polyphenols present in plants, which can prevent the activity of 3FTx by binding to the toxin itself, thus blocking their interaction with their respective receptors [52]. Epigallocatechin-3-gallate (EGCG), a polyphenol, successfully blocked the activity of alpha cobra toxin by binding to the toxin, thereby preventing adherence to the acetylcholine receptor [52]. When the toxin fails to bind to the receptor, minimal neurotoxicity, cardiotoxicity, anticoagulation, and cytotoxicity occurs. Hence, by identifying 3FTx as one of the antigenic proteins, we can target this protein family in developing therapeutics for snake envenomation.

### 4.4 Common antigenic proteins in pit vipers

The common antigenic proteins identified across both pit vipers in this study are SVMP and snaclecs, which align with previous investigations on viper envenomation effects [53,54]. Viper venom induces hemotoxic and myotoxic effects in the victims. The major proteins responsible for these clinical manifestations include PLA_2,_ which causes local inflammation; metalloprotease, leading to haemorrhaging and degradation of fibrinogen; and snaclecs, inhibiting platelet activation [55]. Viperid venoms typically have a relatively long half-life, potentially prolonging patient recovery [54,56]. These proteins are crucial in causing the symptoms observed in victims’ post-envenomation.

#### 4.4.1 Snake C-type lectins (Snaclecs)

Snaclecs have a basic heterodimeric structure with two subunits α and β [57], such as rhodocetin, and both subunits were separately identified in the LC-MS/MS analysis. In our present study, snaclecs were exclusively identified in the viper samples of *C. rhodostoma* and *C. purpureomaculatus*. In the *C. rhodostoma* sample, only snaclec rhodocetin subunit beta was identified in the interaction against CAV, MKAV and BKAV. In contrast, both subunits were identified in the interactions against MPAV, RPAV and HPAV. The snaclec protein from *C. rhodostoma* has dual effects on platelet aggregation. Rhodocetin (four subunits) is a platelet aggregation inhibitor, while rhodocytin (two subunits) induces it. For *C. purpureomaculatus* samples, the interaction against CAV and HPAV only identified snaclec rhodocetin subunit beta. In contrast, both subunits were found to be antigenic in the reaction against MPAV and MKAV. This protein is a potent inhibitor of collagen-induced platelet aggregation; it binds to an integrin α2A domain, blocking collagen from binding to the integrin [58]. It is possible that some antibodies could only bind to the beta subunits of this protein, but antibodies from other antivenom could bind to the alpha subunit of Snaclecs. This could be due to difference the structure of the subunit or the position of the subunits itself.

Currently, there are no small molecules or drugs specifically targeting snaclec rhodocetin or snaclec rhodocytin. Theoretically, an inhibitor for snaclec rhodocetin could allow platelets to adhere to each other and form a haemostatic plug, thereby preventing bleeding that may occur during envenomation. This inhibitor could target the protein or its binding site, integrin alpha2A domain. Similarly, a rhodocytin inhibitor could prevent platelet aggregation by potentially binding to the protein or its specific binding site, C-type lectin domain family 1 member B (CLEC1B/CLEC2). Inhibiting the activities of rhodocytin could prevent the formation of blood clots during envenomation.

### 4.5 Common antigenic proteins in cobras and pit vipers

In this study, we examined the crude venom from two distinct families, each associated with different clinical manifestations following envenomation. Despite this divergence, we have successfully identified common antigenic proteins across both families, notably LAAO and PLA_2_. While LAAO is more prevalent in cobras, viper venom also contains trace amounts of this enzyme [59]. Conversely, PLA_2_ constitutes a sizeable portion of cobra venom composition. Despite the difference in symptoms, pit vipers and cobras share common antigenic proteins, this could be because snake venoms' composition and function can differ between inter and intra-species [31]. Recognising these shared antigenic proteins is crucial, as it provides insights that could assist the development of novel treatment approaches for envenomation cases.

#### 4.5.1 L-amino acid oxidase (LAAO)

Snake venom LAAO poses significant harm to the human body, inducing red blood cell haemolysis and extensive bleeding by compromising blood vessel integrity, leading to haemorrhage [60]. LAAO are also capable of inducing programmed cell death in various cells and tissues resulting in severe organ damage. LAAOs also induce programmed cell death in various tissues, resulting in severe organ damage and increased vascular permeability, leading to fluid leakage into interstitial spaces [60]. Variants like TM-LAAO from *Cryptelytrops mucrosquamatus* and LAAO from *O. hannah* have been reported to be edematogenic [61]. Although the precise mechanism of LAAO-induced oedema remains unclear, it likely differs from typical toxin-mediated inflammatory mediator release, as antihistamines do not alleviate its effects [62]. LAAO triggers local effects such as swelling and necrosis and systemic effects like disseminated intravascular coagulation (DIC) and haemorrhaging, which are consistent with king cobra envenomation symptoms.

Given the conserved structure of LAAO, inhibitors targeting its binding site can be designed using its protein sequence. Aristolochic acid from Aristolochia indica has shown inhibitory effects on LAAO from Russell's viper venom by inducing cell genotoxicity [63]. Subsequently, derivatives of aristolochic acid were synthesised to reduce toxicity while retaining inhibitory activity against LAAO. These derivatives significantly decreased reactive oxygen species induced by LAAO without affecting DNA [64]. Therefore, Inhibiting LAAO holds significant promise in antivenom development due to its crucial role in venom-induced pathogenesis. By targeting LAAO, it's possible to mitigate the destructive effects of snake envenomation, such as haemolysis, bleeding, tissue necrosis, and systemic complications like DIC.

#### 4.5.2 Phospholipase A_2_ (PLA_2_)

PLA_2_ was identified in two of *N. sumatrana* interactions (RPAV and CAV), and in all the pit viper samples. However, the PLA_2_ found in the elapids is a neutral PLA_2_ muscarinic inhibitor. This specific snake venom PLA_2_ inhibits the muscarinic acetylcholine receptors (mAChR) and catalyses the calcium-dependent hydrolysis of 2-acyl groups (amides and esters) in 3-sn-phosphoglyceride [65]. While this protein shares high homology with PLA2 from the *Naja* family, it is the first to be identified as a muscarinic inhibitor [65]. In contrast, the functional role of PLA_2_ identified in pit vipers remains to be elucidated. Nevertheless, PLA_2_ generally exerts diverse toxic effects, which can be categorized into three main groups: neurotoxins, myotoxins, and haemostasis-impairing toxins [66].

Given the wide range of toxic effects attributed to PLA_2_, targeting this compound in snake venom is crucial for developing effective treatments. PLA_2_ inhibitors have been investigated extensively, including compounds derived from plant extracts, steroids, synthetic molecules, and phenolic compounds [67]. For example, the aqueous extract of Casearia sylvestris has demonstrated protective effects against PLA2-induced damage from several snake species, *including Bothrops moojeni, B. pirajai, B. neuwiedi*, and *B. jararacussu* [68,69]. Additionally, synthetic inhibitors like Edunol, a derivative of isoflavonoids, have exhibited anti-PLA_2_ activities against *B. jararacussu* venom [70,71]. Further research in this area holds promise for developing potent PLA_2_ inhibitors for treating snake envenomation.

#### 4.5.3 Snake venom metalloproteinase (SVMP)

SVMP are zinc-dependent enzymes predominantly found in vipers but also present in elapids, play a crucial role in snake venom toxicity. This study identified SVMP as antigenic in both vipers and king cobra. These enzymes can be classified into three classes based on size and structure [72]. The P-I class has a single catalytic metalloproteinase domain and exhibits less severe toxic effects [73]. In contrast, the P-II class has an additional disintegrin domain, influencing its integrin-binding motif and domain composition [72]. The effects of the P-II proteins heavily depend on the integrin-binding motif and domain composition [72]. Lastly, the P-III class contains metalloproteinase, disintegrin-like, and cysteine-rich domains, contributing to potent haemorrhagic activities. The variety of structure in P-III class is due to proteolytic cleavage, occurrence of other ancillary domains and continual domain loss [72,74].

The LC-MS/MS analysis from the present study (Table 2, Table 5, Table 6) revealed the presence of SVMP with a disintegrin, (Zinc metalloproteinase-disintegrin-like ohanin, Zinc metalloproteinase/disintegrin, Snake venom metalloproteinase kistomin) suggesting membership in the SVMP P-II subclass. Zinc metalloproteinase-disintegrin-like ohanin inhibits adenosine diphosphate (ADP), disrupting the platelet aggregation [75]. Zinc metalloproteinase/disintegrin from *C. rhodostoma* is also known as snake venom metalloproteinase rhodostoxin and it impairs the envenomed victims’ homeostasis [76]. Snake venom metalloproteinase kistomin from *C. rhodostoma* also inhibits the platelet aggregation by blocking the adhesion of platelets immobilised collagen [77].

Neutralising the haemorrhagic effects of SVMPs is crucial in snakebite treatment. Antibodies, small molecules, or protease inhibitors targeting these proteins can help mitigate their effects. Matrix metalloprotease inhibitors (MMPIs), such as marimastat and batimastat, have shown promise in reducing haemorrhage, necrosis, and oedema induced by SVMPs [78]. Marimastat inhibits lethal and haemorrhagic effects from *E. ocellatus* venom, while batimastat potentially treats local haemorrhage and dermonecrosis post-*Bothrops asper* envenomation [79,80]. Further research in this area is essential for developing effective treatments for snake envenomation.

## 5 Conclusion

In this study, we successfully identified the antigenic proteins in the venoms of five snake species By using immunoprecipitation coupled with LC-MS/MS, we identified key antigenic proteins from pit vipers and cobras, revealing distinct venom components such as 3FTXs in cobras and Snaclecs in pit vipers. The presence of common proteins, including LAAO, PLA_2_, and SVMP, across both families suggests potential targets for developing broader spectrum antivenoms. Current findings from the conventional antivenoms primarily target antigenic toxins; however, proteins not recognized by these antivenoms pose a challenge for developing new treatments. It is also important to note that several antigenic proteins do not necessarily cause clinical manifestations, adding complexity to the development of effective therapeutic interventions. The immunoprecipitation assay, despite its advantages in reproducibility and cost-efficiency, has potential cross-contamination risks, emphasizing the need for careful dissection and sample handling. Nonetheless, by understanding the specific toxic antigenic proteins involved in snake envenomation, we can potentially enhance the efficacy and safety of snakebite therapy. Further research is essential for advancing snakebite management and reducing the global burden of snakebite-related morbidity and mortality.

## Funding

This work was supported by theMalaysia 10.13039/501100002385Ministry of Higher Education, Fundamental Research Grant Scheme (FRGS) 2022 (Ref: FRGS/1/2022/SKK10/MUSM/03/2), the 10.13039/501100000683Royal Society of Tropical Medicine and Hygiene (RSTMH) Early Career Grant Award 2020 and 10.13039/501100021809Jeffrey Cheah School of Medicine and Health Sciences, Monash University Malaysia, 2020 Seed Grant.

## Data availability statement

The data has not been deposited into a publicly available repository. The data is included in the article/supplementary material/referenced in the article.

## CRediT authorship contribution statement

**Preetha Rajendiran:** Writing – original draft, Methodology, Investigation, Formal analysis, Data curation. **Rakesh Naidu:** Writing – review & editing, Validation, Supervision. **Iekhsan Othman:** Validation, Supervision. **Syafiq Asnawi Zainal Abidin:** Writing – review & editing, Validation, Supervision, Project administration, Funding acquisition, Formal analysis, Conceptualization.

## Declaration of competing interest

The authors declare the following financial interests/personal relationships which may be considered as potential competing interests:

Syafiq Asnawi Zainal Abidin reports financial support was provided byMalaysia 10.13039/501100002385Ministry of Higher Education. Syafiq Asnawi Zainal Abidin reports financial support was provided by 10.13039/501100000683Royal Society of Tropical Medicine and Hygiene. Syafiq Asnawi Zainal Abidin reports financial support was provided by 10.13039/501100010699Monash University Malaysia 10.13039/501100021809Jeffrey Cheah School of Medicine and Health Sciences. If there are other authors, they declare that they have no known competing financial interests or personal relationships that could have appeared to influence the work reported in this paper.
